# Strain Response and Buckling Behavior of Composite Cylindrical Shells Subjected to External Pressure with One End Fixed and the Other under Free Boundary Conditions

**DOI:** 10.3390/s22186781

**Published:** 2022-09-08

**Authors:** Ke-Chun Shen, Xue-Jian Liu, Yi-Hua Huang, Guang Pan

**Affiliations:** 1 Structural Ceramics and Composites Engineering Research Center, Shanghai Institute of Ceramics, Chinese Academy of Sciences, Shanghai 200050, China; 2 School of Marine Science and Technology, Northwestern Polytechnical University, Xi’an 710072, China; 3 Key Laboratory for Unmanned Underwater Vehicle, Northwestern Polytechnical University, Xi’an 710072, China

**Keywords:** strain response, buckling behavior, boundary conditions, hydrostatic pressure

## Abstract

This study aims to reveal the buckling behavior of filament-wound composite cylindrical shells subjected to external pressure. The boundary conditions of the cylindrical shells were one end fixed and the other free. The carbon fiber stacking sequences were [±90]_2_/([±20]/[±90]/[±40]/[±90]/[±60]/[±90])_2_/[±90]. Finite element software ANSYS 16.2 was used for the numerical simulation to predict the critical buckling pressure and buckling behavior of composite cylindrical shell. External hydrostatic pressure tests were conducted, where the buckling behavior and strain response were observed. Numerical simulation accurately predicted the critical buckling pressure of carbon fiber/epoxy filament composite cylindrical shells under external pressure with 3.5% deviation from the experimental results. The buckling modes simulated by the finite element method agreed well with the deformed shape observed in the experiment, which was characterized by the uniform distribution of the three hoop waves. Comparing the axial compressive strain and hoop compressive strain of the composite shell, it was found that the circumferential stiffness of the shell was weaker than the axial stiffness. In addition, a comparative study of the strains of the fixed-end and free-end metal control sleeves was carried out. The results show that the boundary conditions have a significant influence on the strain response of control sleeves.

## 1. Introduction

Buckling [1,2,3,4] is a major failure form of thin walled structures under external hydrostatic pressure. The submersible pressure shell structure has been made of traditional metal materials such as high-strength steel, titanium alloy, and aluminum alloy. Generally, submarines require a large buoyancy reserve capacity because functional equipment and switchable loads need to be carried. Therefore, lightweight materials with high mechanical properties are expected to apply in submarine structures. Fiber composite materials have high specific strength and specific stiffness [5,6]. Filament-wound composite cylindrical shells have been widely used in underwater vehicles under external hydrostatic pressure, aiming to improve the buoyancy-to-weight ratio of the vehicle. Composite materials have good sound absorption properties, which can reduce the danger of underwater vehicle being detected by sonar equipment [7]. In addition, the stability of composite materials to chemical reactions can improve the corrosion resistance of submarine structures [8].

Numerous studies have focused on the analytical solution of shell buckling of composite cylinder subjected to external hydrostatic pressure. Cho [9] proposed an empirical formula to predict the ultimate load capacity of composite cylinder under external hydrostatic pressure taking into account both structural buckling and material failure. Imran [10] investigated the linear buckling of composite cylinder with various geometric sizes and layup configurations under external pressure. Based on the classical laminated plate theory, Ehsani [11] and Cai [12] obtained the elastic buckling load of laminated composite structures. Besides, many researches [13,14,15,16] evaluated the buckling failure of composite structures using finite element analysis.

In order to maximize the critical buckling pressure of composite cylinder subjected to external hydrostatic pressure, optimization design [17,18,19,20,21,22,23,24] has been studied for composite cylindrical shells, where the fiber orientation and stacking sequence were selected as optimization variables. Some researchers [25,26,27,28,29] studied the buckling phenomenon of composite cylinder under external hydrostatic pressure by experiment method. Carvelli [1] found large deformation of glass fiber reinforced polymer cylindrical shell in the hydrostatic pressure test. Hur [30] conducted experiment and numerical simulation to study the buckling and post-buckling of composite cylindrical shell under external pressure. Moon [31] investigated the critical buckling and failure characteristics of filament-wound carbon/epoxy composite cylinder with [±30/90]_FW_, [±45/90]_FW_ and [±60/90]_FW_ winding sequences subjected to external hydrostatic pressure. Usually the ends of composite cylindrical shell were bonded with the metal control sleeves, and they formed a glued interface where strength was most critical in these regions when the shell was subjected to hydrostatic pressure. Reviewing the above studies, research progress on the strain response of the interface was rarely reported.

This paper focused on the strain response and buckling behavior of composite cylinder under hydrostatic pressure with one end fixed and the other free. Three carbon fiber/epoxy filament composite cylindrical shells were test. The strain responses of composite cylinder, control sleeves with fixed/free boundary conditions were measured. Shell deformation and buckling behavior were observed. Numerical simulation method was also used for comparison of the critical buckling pressure and buckling modes.

## 2. Specimen and External Hydrostatic Pressure Test

**Figure 1:**
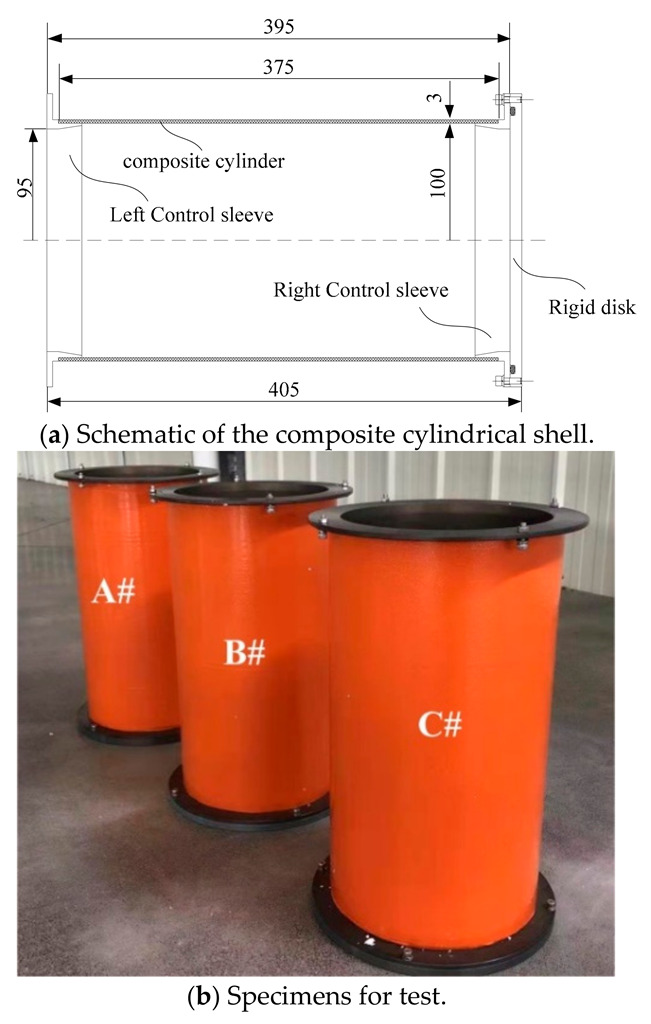
Composite cylindrical shells.

The specimens were made of Carbon fiber (T700-12K) /epoxy by a filament winding process with [±90]_2_/([±20]/[±90]/[±40]/[±90]/[±60]/[±90])_2_/[±90] sequence. The type of epoxy resin is E51. The curing agent is Methyltetrahydrophthalic anhydride. All the cylinders have a 100 mm inner radius, a 375 mm axial length and a 3 mm thickness (see Figure 1a). The composite cylinder was bonded with two metal control sleeves (Aluminum alloy 7075: *E* = 71 GPa, *ν* = 0.33). The cylindrical shell was closed by a rigid disk (Aluminum alloy 7075: *E* = 71 GPa, *ν* = 0.33) at its end. A total of three specimens (see Figure 1b) were tested to investigate the buckling behavior of composite cylindrical shell subjected to external hydrostatic pressure. The mechanical properties of Carbon fiber (T700-12K)/epoxy were listed in Table 1.

**Figure 2:**
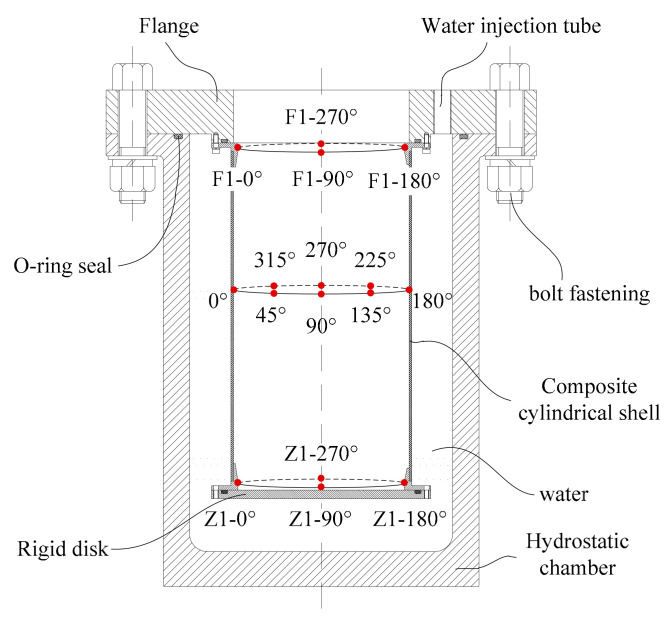
Test set-up and locations of strain gauges.

A schematic diagram of the test setup was shown in Figure 2. The flange was connected with the cylindrical shell and the static pressure chamber, which formed a closed space. The cylindrical shell was fixed on the flange with a hole, so the inner wall of the cylindrical shell can be observed from above the flange. To measure the strain response of the control sleeve, four resistance strain gauges were glued to the inner wall of the left control sleeve at 0°, 90°, 180°, and 270°. The strain gauge can measure the strain in two directions, one is the axial direction of the shell, and the other is the hoop direction of the shell. The resistance value of the strain gauge is 120 Ω. As for the distribution of the strain gauges on the right end control sleeve, it was similar to that of the left control sleeve. Additionally, six strain gauges were glued to the inner wall of the composite cylinder at 0°, 45°, 90°, 135°, 180°, 225°, 270° and 315° in the middle length. Water was continuously injected into the hydrostatic chamber through the injection pipe using a high-pressure pump. Thus, the specimens experienced axial pressure on the end rigid disk and lateral pressure on the composite cylindrical shell.

## 3. Numerical Simulation

**Figure 3:**
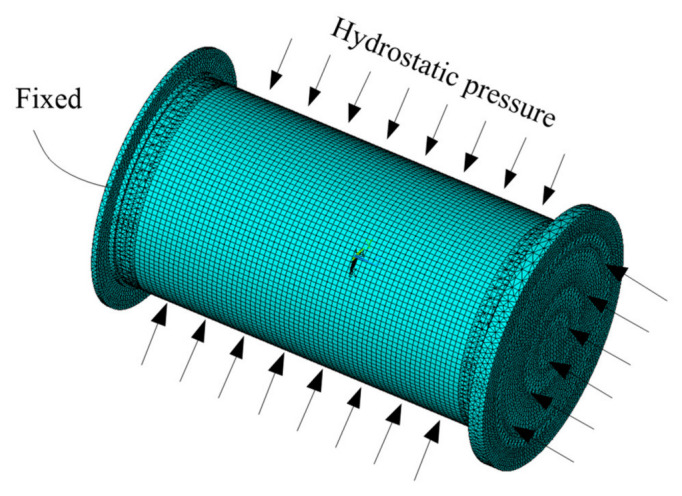
Finite element model and boundary conditions.

The buckling behavior of composite cylindrical shells subjected to external hydrostatic pressure with one end fixed and the other free boundary conditions were analyzed by numerical simulation. Static analysis and linear buckling analyses were performed in sequence to predict the critical buckling load by finite element software ANSYS 16.2. A three-dimensional structural element SHELL 281 was used to mesh the composite cylindrical shell. SHELL 281 has eight nodes. Each node has six degrees of freedom involving translation in the three axes (*x*, *y* and *z* axes) and rotation about the three axes. As for the other parts of the cylinder, a three-dimensional tetrahedral structural solid element SOLID187 was used to mesh the rigid end disk and control sleeves. SOLID187 has ten nodes. Each node has three degrees of freedom involving translation in the three axes. For specimen used for test, the control sleeves were bonded to the composite cylindrical shell. To analyze the bonding state, the inner surface of the composite cylindrical shell was simulated by the target surface using TARGE170 element, and the outer surface of the control sleeves was simulated by the contact surface using CONTA174 element. The boundary conditions in the numerical simulation were consistent with those in the experiment. The left control sleeve was fixed, and the other end of the structural was free. The load condition was that the structure was subjected to external hydrostatic pressure (see Figure 3).

## 4. Results and Discussion

### 4.1. Buckling Pressure and Buckling Mode

**Figure 4:**
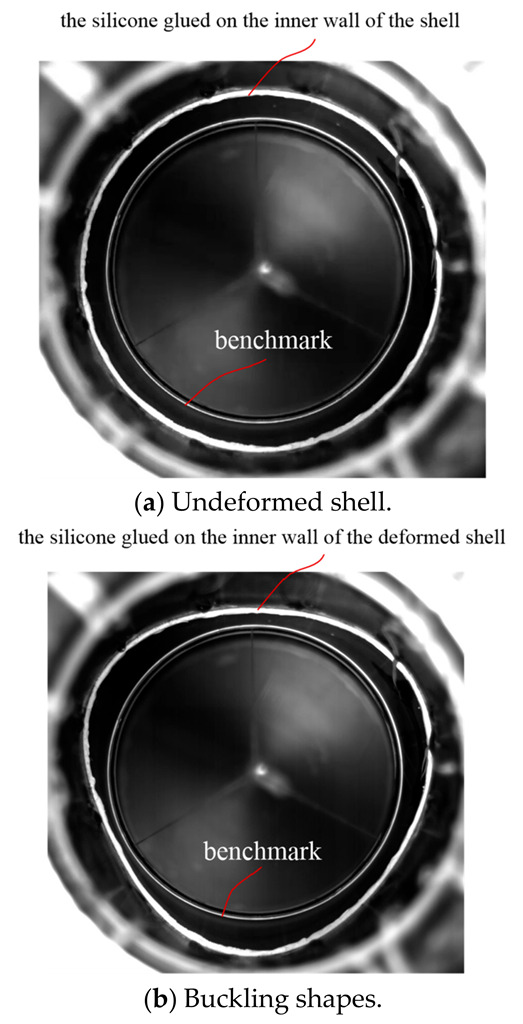
Deformation behavior (A#) observed in test.

**Figure 5:**
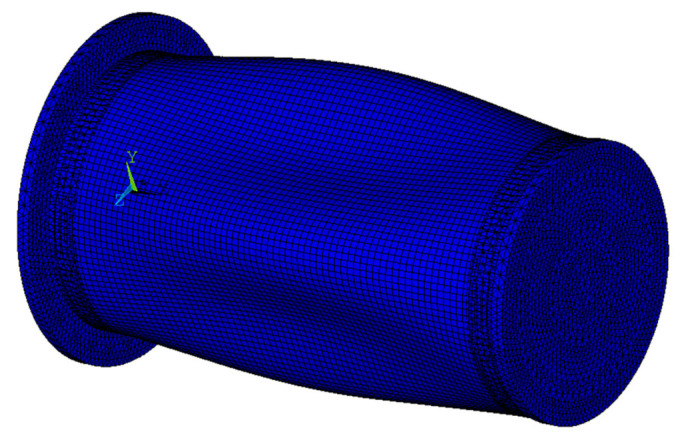
Buckling modes predicted by FEM.

Both the experimental and numerical simulation results were listed in Table 2. The experimental buckling pressure of the A#, B# and C# cylinders are 3.06 MPa, 3.12 MPa and 3.09 MPa, respectively. The deviations of the predicted buckling pressures of the A#, B# and C# cylinders from the experimental results were 1.31%, 3.21% and 2.27%, respectively. In the experiment, a high-speed camera was used to observe the large deformation of the shell. Figure 4 show the structural morphology of the undeformed (Figure 4a) and buckling shape (Figure 4b). There are two white circles in Figure 4; the larger circle represents the silicone glued on the inner wall of the shell, and the smaller circle is the benchmark. By comparing the small circle with the deformed large circle, the peaks and troughs can be distinguished clearly. As for the buckling modes, three waves were clearly visible and evenly distributed in the circumferential direction according to the experimental observation (Figure 4b) and finite element prediction (Figure 5). Comparing the numerical simulation and test results, it was shown that the numerical method accurately calculated the buckling pressure, and reasonably predicted the buckling mode of filament-wound composite cylindrical shell under external hydrostatic pressure.

### 4.2. Strain Response of Control Sleeve

**Figure 6:**
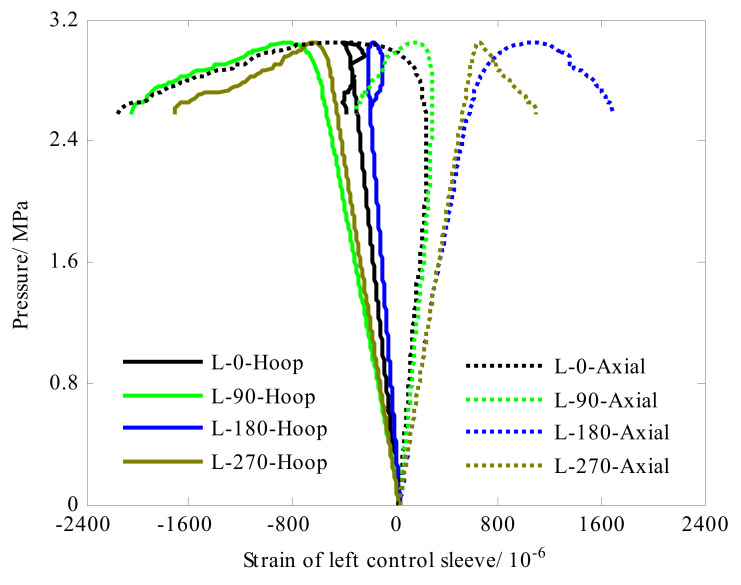
Strain response of the left control sleeve of cylinder A#.

**Figure 7:**
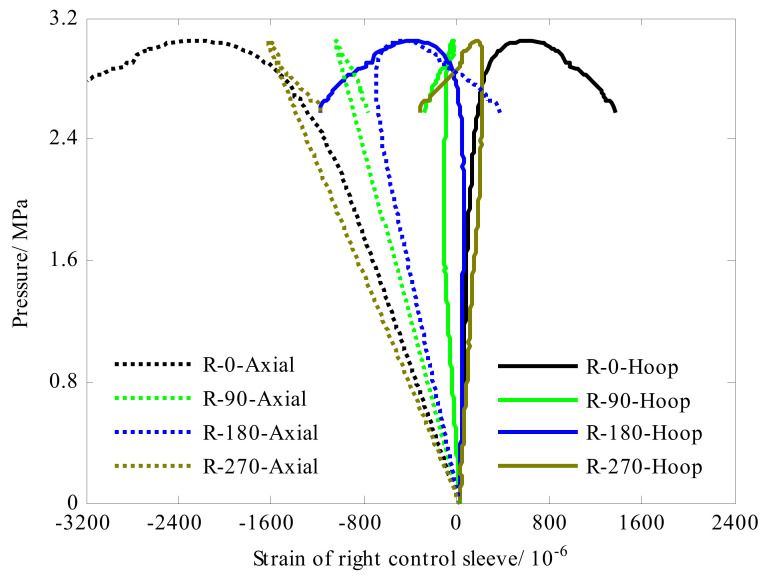
Strain response of the right control sleeve of cylinder A#.

Eight strain gauges were evenly attached along the circumference (0°, 90°, 180° and 270°) in the left and right control sleeves, respectively. Each strain gauge can measure axial strain and hoop strain. The strain responses of the left control sleeve were shown in Figure 6. Under the compressive load of the hydrostatic pressure environment, in axial direction the left control sleeve exhibited positive strain, and in hoop direction it presented negative strain. As for the right control sleeve (see Figure 7), it was interesting that the strain response exhibited negative strain in axial direction and positive strain in hoop direction. This phenomenon can be attributed to the difference in boundary conditions in both ends. For the boundary condition at the left end, it was fixed and connected to the flange of the hydrostatic pressure. Under the action of hydrostatic side pressure, the left control sleeve was compressed circumferentially. Considering the isotropic characteristics of metal materials, the hoop compression of the skirt would cause axial elongation, so that the strain response of the skirt presented hoop compression and axial tension. For the boundary condition at the right end, under the action of axial pressure, the rigid end disk flexed inward, causing additional bending moment. The additional bending moment was transferred to the right control sleeve, where the circumferential expansion occurred. Taking the isotropic characteristics of metal materials into account, expansion in the circumferential direction would cause axial compression of the right control sleeve. In the test, the critical buckling load of the composite cylindrical shell was 3.06 MPa. As shown in Figure 6 and Figure 7, strain response of control sleeve was basically linear with the external load until the shell collapsed.

### 4.3. Strain Response of Composite Cylindrical Shell

**Figure 8:**
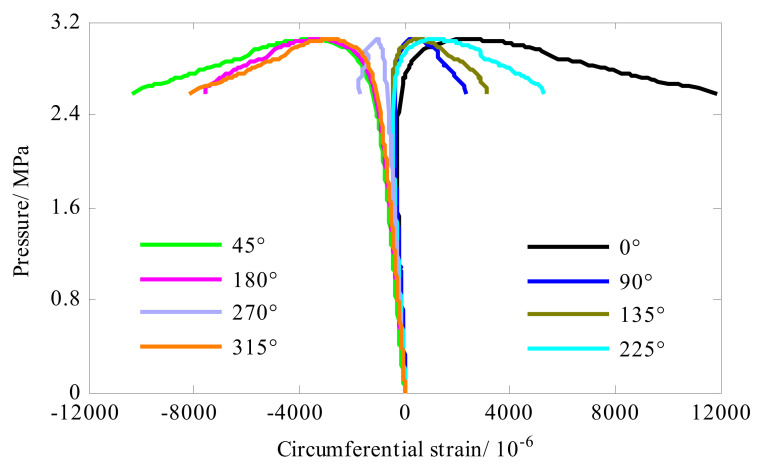
Strain response of the composite cylindrical shell A#.

**Figure 9:**
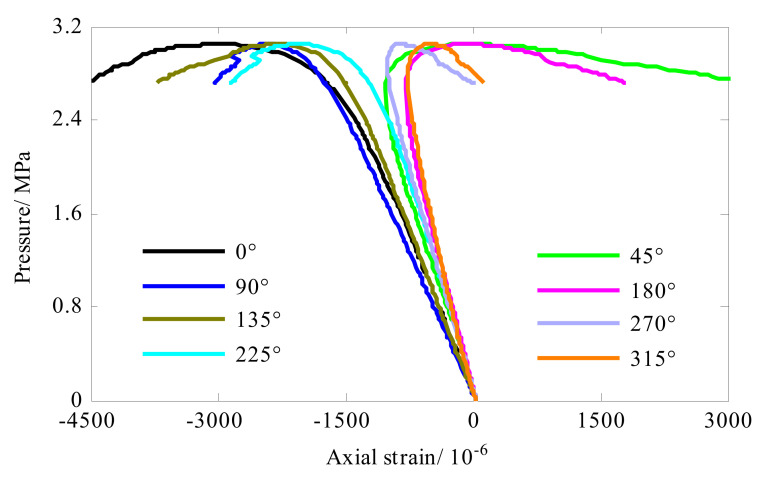
Strain response of the composite cylindrical shell A#.

**Figure 10:**
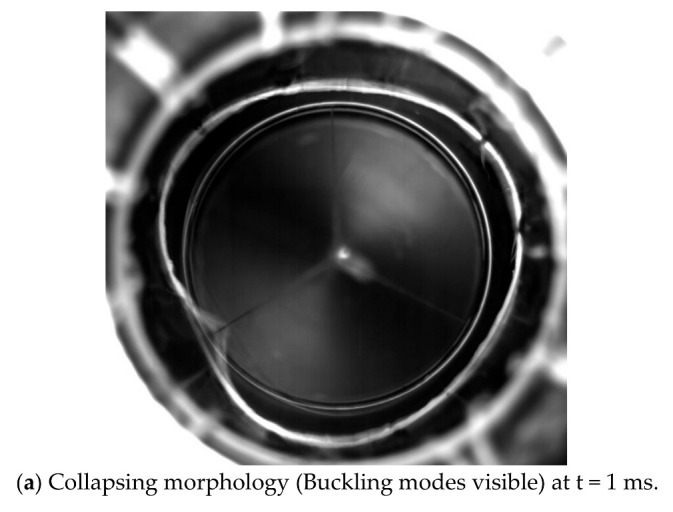

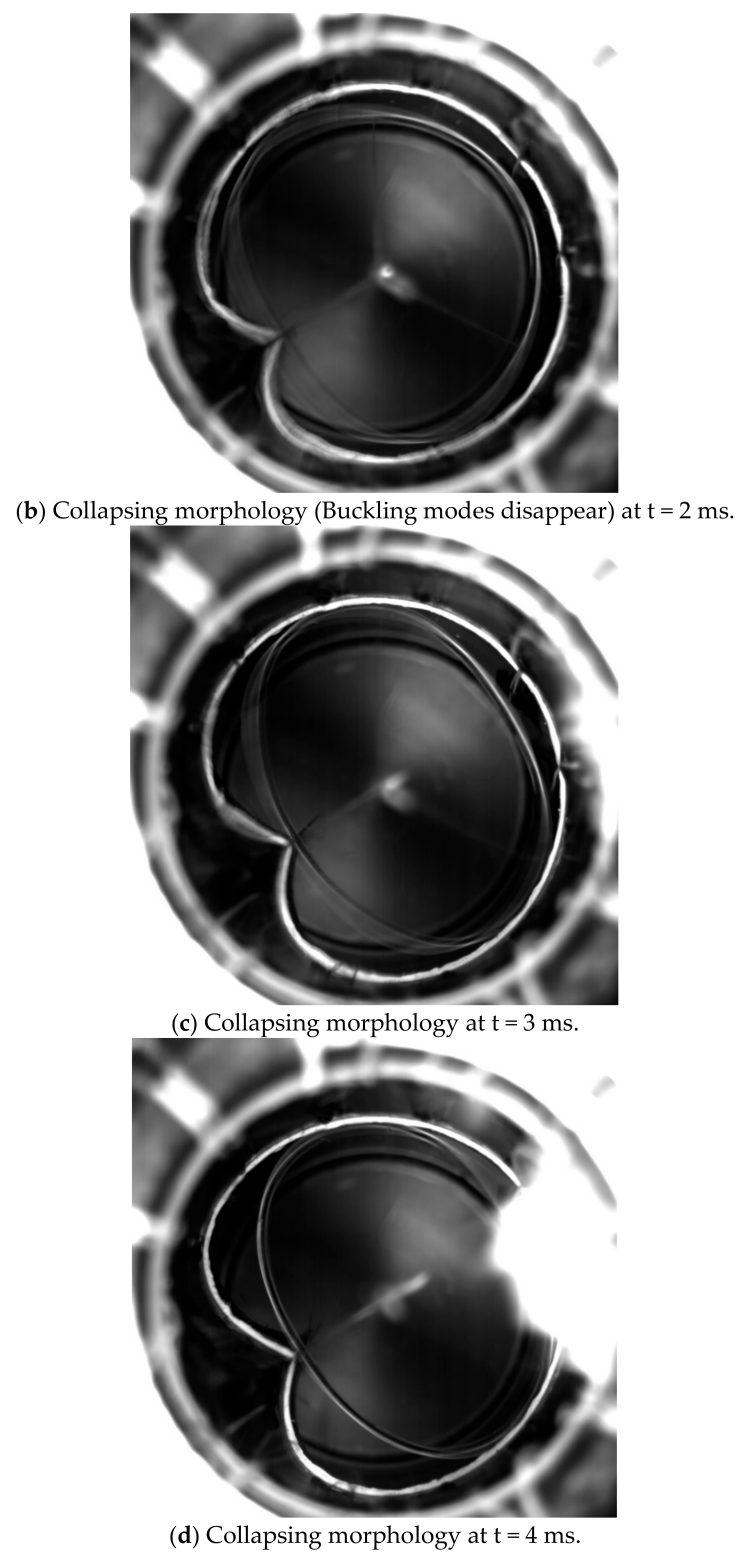
Failure of the collapse moment.

In the middle of the length of the shell, eight strain gauges were distributed evenly along the circumference direction (0°, 45°, 90°, 135°, 180°, 225°, 270° and 315°). Each strain gauge can measure axial strain and hoop strain. The axial strain and hoop strain of the composite cylindrical shell were shown in Figure 8 and Figure 9, respectively. Before the shell buckled, the strain response was linear with the load and presented compressive. As the external pressure approached the buckling load, the strain response gradually became nonlinear. Then, the bearing capacity of the shell gradually decreased. For circumferential strain (see Figure 8), the strain of measuring points (0°, 90°, 135° and 225°) changed from compressive strain to tensile strain. The strain value of the remaining measuring points continued to increase until the shell collapsed. That was to say, when the shell entered the post-buckling stage, the strain bearing capacity was weakened, and strain response in hoop direction of some measuring points (0°, 90°, 135° and 225°) was reversed. For axial strain (see Figure 9), strain reversal occurred at 45°, 180°, 270° and 315° locations. In summary, in the post-buckling stage, the strain reversal occurred at all the measuring points, some of the measuring points showed the axial strain reversal, and the remaining measuring points present the hoop strain reversal. Additionally, comparing Figure 9 and Figure 10, it was found that the axial compressive strain of the shell was significantly smaller than the hoop compressive strain in the linear stage. This meant that the axial stiffness of the shell was greater than the hoop stiffness.

The collapsing morphology observed in test was shown in Figure 10. At the moment of collapse (see Figure 10a at t = 1 ms), the buckling mode of the composite cylindrical shell was visible. In the next instant (see Figure 10b at t = 2 ms), the buckling mode of the shell disappeared, and the shell returned to its original shape. Immediately after, water rushed into the composite shell (see Figure 10c,d at t = 3 ms t = 4 ms). In the extremely short time, the pressure in the static pressure chamber dropped sharply. The crushing positions of the three composite cylindrical shells have a common feature, that is, they are all located at the troughs in the buckling waveform. The circumferential stiffness of the shell was inherently weak, and after the buckling and large deformation, the circumferential wave trough further reduced the circumferential bending stiffness of the shell. Therefore, in the post-buckling stage, the bearing capacity of the shell gradually decreased, and strength failure occurred at the trough. Subsequently, the failure extends to both ends, forming an axial crack.

## 5. Conclusions

Aiming to reveal the buckling behavior of filament-wound composite cylindrical shells subjected to external pressure, three composite cylinders were tested to ultimate collapse. Numerical simulation was performed to predict the buckling load. The following results are summarized:

(1) Numerical simulation method calculated the critical buckling load within 3.5% deviation from test results, not taking the initial imperfections of the cylinders into consideration. As for the buckling modes, predictions given by numerical method were in good agreement with the phenomena observed in tests, which were characterized by three waves in the hoop direction.
(2) In the static deformation stage, the magnitudes of compressive strains of the composite cylinder in the axial direction were smaller than those in hoop direction. It revealed that the axial stiffness of the shell was greater than the hoop stiffness.
(3) Boundary conditions had a significant influence on the strain response of the control sleeves of the shell. For the left control sleeve, it presented positive strains in axial direction and negative strain in hoop direction. For the right control sleeve, it showed negative strain in the axial direction and positive strains in the hoop direction.

## Figures and Tables

**Table 1:** Properties of Carbon fiber (T700-12K) /epoxy.

Properties	Symbol	Value	Unit
Elastic modulus	***E***_11_	102	GPa
	***E***_22_	7	GPa
	***E***_33_	7	GPa
Poisson’s ratio	***v***_12_	0.16	
	***v***_13_	0.16	
	***v***_23_	0.32	
Shear modulus	***G***_12_	8	GPa
	***G***_13_	8	GPa
	***G***_23_	4.5	GPa

**Table 2:** Experimental and numerical simulation results.

ID	Stacking Sequence	Buckling Pressure/MPa	
		Test	FEM (Error, %)
A#	[±90]_2_/([±20]/[±90]/[±40]/[±90]/[±60]/[±90])_2_/[±90]	3.06	3.02 (1.31%)
B#		3.12	3.02 (3.21%)
C#		3.09	3.02 (2.27)

## Data Availability

Not applicable.
